# Developmental genes targeted for epigenetic variation between twin-twin transfusion syndrome children

**DOI:** 10.1186/1868-7083-5-18

**Published:** 2013-10-03

**Authors:** Carmen J Marsit, Devin C Koestler, Debra Watson-Smith, Charlotte M Boney, James F Padbury, Francois Luks

**Affiliations:** 1 Department of Pharmacology and Toxicology, Geisel Medical School at Dartmouth, Hanover, NH 03755, USA; 2 Department of Community and Family Medicine, Section of Epidemiology and Biostatistics, Geisel Medical School at Dartmouth, Hanover, NH 03755, USA; 3 Division of Pediatric Surgery, Fetal Treatment Program of New England, Boston MC, USA; 4 Department of Surgery, Fetal Treatment Program of New England, Boston MC, USA; 5 Division of Endocrinology, Rhode Island Hospital, Providence, RI USA; 6 Department of Pediatrics, Rhode Island Hospital, Providence, RI USA; 7 Program in Fetal Medicine, Warren Alpert Medical School at Brown University, Providence, RI USA; 8 Department of Pediatrics, Women and Infants Hospital, Providence, RI, USA

**Keywords:** Twin-twin transfusion syndrome, Growth restriction, DNA methylation, Intrauterine environment, Fetal

## Abstract

**Background:**

Epigenetic mechanisms are thought to be critical in mediating the role of the intrauterine environment on lifelong health and disease. Twin-twin transfusion syndrome (TTTS) is a rare condition wherein fetuses share the placenta and develop vascular anastomoses, which allow blood to flow between the fetuses. The unequal flow results in reciprocal hypo- and hypervolemia in the affected twins, striking growth differences and physiologic adaptations in response to this significant stressor. The donor twin in the TTTS syndrome can be profoundly growth restricted and there is likely a nutritional imbalance between the twins. The consequences of TTTS on fetal programming are unknown. This condition can now be effectively treated through the use of fetal laparoscopic procedures, but the potential for lifelong morbidity related to this condition during development is apparent. As this condition and the resulting uteroplacental discordance can play a role in the epigenetic process, we sought to investigate the DNA methylation profiles of childhood survivors of TTTS (n = 14). We focused on differences in both global measures and genome-wide CpG specific DNA methylation between donor and recipient children in this pilot study in order to generate hypotheses for further research.

**Results:**

We identified significant hypomethylation of the LINE1 repetitive element in the peripheral blood of donor children and subtle variation in the genome-wide profiles of CpG specific methylation most prominent at CpG sites which are targets for polycomb group repressive complexes.

**Conclusions:**

These preliminary results suggest that coordinated epigenetic alterations result from the intrauterine environment experienced by infants with TTTS and may, at least in part, be responsible for downstream health conditions experienced by individuals surviving this condition.

## Background

Epidemiologic studies have clearly linked infant growth to antenatal environmental factors, including diet, xenobiotic exposures, stress and lifestyle factors, as a significant risk factor for long-term chronic disease, particularly cardiovascular disease and metabolic syndromes [1-3]. These studies, and a growing literature on the role of development on lifelong health, would suggest that a significant proportion of disease risk can be linked to the intrauterine environment.

On a molecular level, epigenetic mechanisms have been invoked to explain mechanistically how experiences during a narrow but critical and susceptible period of time can influence long-term processes leading to health and disease. DNA methylation, a key epigenetic mechanism, is a clear focus of studies on the developmental origins of health and disease. It represents a stable modification of DNA that can be propagated during cell division, yet is susceptible to environmental influence. This is particularly true during development when the cell-specific patterns of methylation that define cellular differentiation are set [4-6]. Studies of specific candidate genes as well as genome-wide examinations of DNA methylation have demonstrated clear relationships to infant birthweight, supporting the role of DNA methylation in mediating these risks [7,8]. This is particularly relevant to correlations in genes involved in metabolism, growth and cardiovascular disease [9].

Studies of twins represent powerful approaches to understanding the importance of shared or disparate environment as well as the contribution of genetics on phenotype [10]. These studies also represent a powerful opportunity to examine the impact of the environment on epigenetic phenomena and, in turn, the contribution of epigenetics on various health outcomes [11,12]. Twin studies are powerful in interrogating both the intrauterine and the postnatal environment, as generally monozygotic, and to a slightly lesser degree, dizygotic twins share the same intrauterine environment. Nonetheless, to date, most studies of twin epigenetics have focused on older and adult cohorts, ignoring the opportunity to consider the role of the intrauterine environment [13-17].

Twin-twin transfusion syndrome (TTTS) patients offer a unique opportunity to investigate how an adverse intrauterine environment compares to a rich environment, on DNA methylation and, potentially, on later health outcomes while controlling for genetic contribution. Approximately 70% of monozygotic twins are monochorionic and diamniotic (DiMo), following embryo division before the post-implantation blastocyst phase. These fetuses share a placenta, and virtually all develop placental vascular anastomoses, which allow blood to flow between the fetuses [18]. In cases of TTTS, which represents approximately 15% of DiMo cases, the blood supply sharing becomes unequal, due to as yet incompletely understood mechanisms that may include the number and/or type of anastomoses[19]. If severe and progressive, TTTS leads to almost 90% perinatal mortality [20]. The ‘donor’ twin becomes chronically hypovolemic and anuric, which leads to severe oligohydramnios. This twin is typically growth restricted as well – a consequence of the progressive cardiac failure, but also of the smaller placental share often associated with the donor. The ‘recipient’ twin becomes polyuric, resulting in severe polyhydramnios. This state can lead to cardiac failure, hydrops and fetal death as well, as chronic hypervolemia leads to (right) ventricular dilation, tricuspid regurgitation and global cardiac dyskinesia [21]. Thus, this syndrome results in a number of distinct pathophysiologic changes in each of the genetically identical twins. Although a number of treatment approaches have been proposed and utilized with varying success to treat this condition, in recent years selective fetoscopic laser photocoagulation of placental vessels has become more widely available and is now considered the only effective treatment in severe, progressive forms of the disease [22-24]. This treatment consists of obliterating all intertwin vascular connections at the placental level, effectively halting the twin-to-twin transfusion and restoring the balance of the twins’ blood supplies. While survival of at least one twin is seen in 80% of cases [25], not all fetuses fully recover. In some, the renal, cardiovascular or neurological effects of the syndrome are already irreversible [26,27]; in addition, unequal placental share seen in some twin pregnancies (a problem that cannot be corrected by fetal surgery) causes persistent placental insufficiency and growth restriction, usually of the donor [28]. In part because of the inherent risks of twin gestations, the chronic morbidity of TTTS and the relative invasiveness of fetal intervention, TTTS pregnancies and infants may also suffer from miscarriage, preterm birth, neonatal intensive care unit admissions, respiratory distress syndrome and intraventricular hemorrhage [29]. Long term morbidity in survivors of TTTS can include renal, cardiovascular and neurodevelopmental deficits [30,31], although a number of studies have suggested that morbidity rates in TTTS cases treated with fetoscopic laser photocoagulation are no different than those observed in similarly preterm populations [32-34]. More subtle long term effects, such as those on metabolic disease and neurobehavioral outcomes, have been less comprehensively evaluated. These effects could be of great interest as a model in defining underlying mechanisms of the developmental origins of these outcomes, including the epigenetic mechanisms potentially responsible. Moreover, the immediately stabilizing effect of fetoscopic laser photocoagulation on the uteroplacental environment may allow us to pinpoint critical time points during gestation when these epigenetic phenomena are critical. Finally, the co-occurrence of small placental share in some donor twins may help us differentiate between adverse uteroplacental factors that are correctible (TTTS) and those that are not (placental insufficiency).

In this study, we examined how DNA methylation profiles in cases of TTTS are impacted by donor-recipient status, a model of extreme adverse and rich intrauterine conditions. This condition occurred during a critical window of development during which DNA methylation patterns are being set. Our aim is to define broadly where variation in DNA methylation is occurring in this situation, as it may aid in defining not only key pathways potentially affected by the intrauterine growth disturbances that result from TTTS but also point to potential long-term consequences worthy of further study.

## Results

The characteristics of the population of children involved in the study are provided in Table 1. Our population consisted of four twin pairs who survived TTTS following corrective surgery, as well as six sole survivors, for a total of 14 subjects. Samples of saliva (n = 14) and peripheral blood (n = 11) were obtained where possible. Current ages of the children ranged from five months to eight years. Birth weights of these children ranged from 380 to 3600 g, with gestational times from 24 to nearly 40 weeks. Fetal laser ablation surgery to repair the anastomoses was performed from 15 to 24 weeks of gestation.

**Table 1:** Characteristics of the population

**ID**	**Twin**	**Donor**-**recipient status**	**Sex**	**Gestational age at laser correction of anastomoses** (**wks**)	**Gestation time to birth** (**wks**)	**Current age** (**y**)	**Birth weight** (**g**)	**Samples provided**
556	604	Donor	M	18.14	29.43	5	917	Blood/saliva
604	556	Recipient	M	18.14	29.43	5	1,315	Blood/saliva
576	663	Recipient	M	20.86	30	3	1,380	Saliva
663	576	Donor	M	20.86	30	3	1,240	Blood/saliva
606	671	Recipient	M	23.86	25.86	6	775	Saliva
671	606	Donor	M	23.86	25.86	6	490	Saliva
614	651	Donor	M	15.86	35.43	0.42	2,700	Blood/saliva
651	614	Recipient	M	15.86	35.43	0.42	3,010	Blood/saliva
573	--	Recipient	F	23.57	24	8	380	Saliva
579	--	Donor	F	22.14	32.57	2	1,870	Blood/saliva
616	--	Donor	F	24.00	37.71	4	2,948	Blood/saliva
632	--	Donor	M	22.86	28.71	5	1,370	Blood/saliva
685	--	Donor	F	22.71	35.14	0.75	2,170	Blood/saliva
692	--	Recipient	F	19.43	39.71	2	3,600	Blood saliva

Total 14 subjects, 14 saliva, 11 blood.

**Figure 1:**
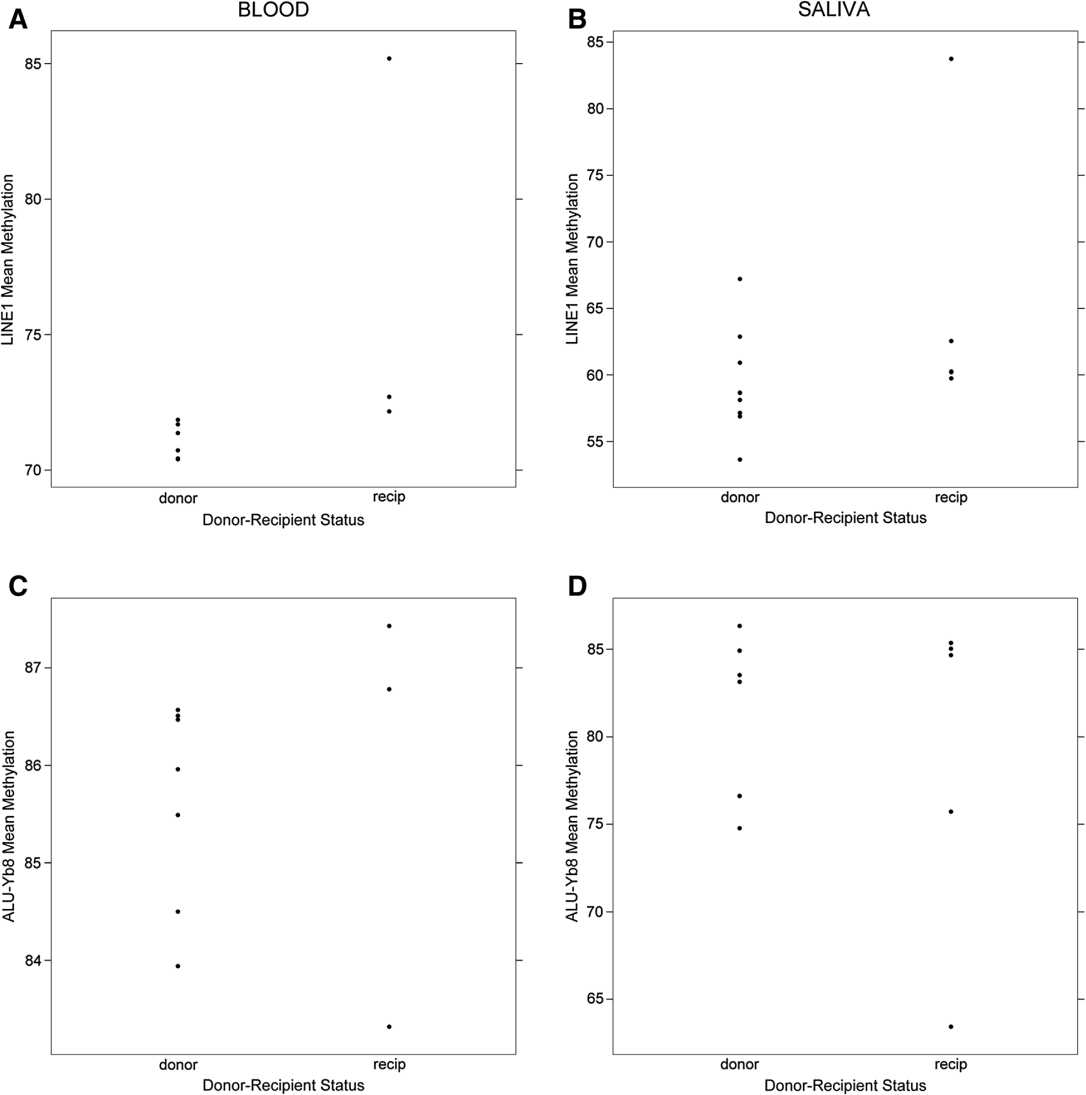
**Extents of repetitive region DNA methylation between donors and recipients stratified by sample type.** Mean LINE1 methylation extent in peripheral blood **(A)** and in saliva **(B)**. Mean ALU-Yb8 methylation extent in peripheral blood **(C)** and saliva **(D)**. The median of the LINE1 methylation extent in peripheral blood between donors and recipients was statistically significantly different (Mann–Whitney U test, *P* <0.03).

Assays to examine the global extent of DNA methylation based on the methylation extents in the LINE1 and ALU-Yb8 repetitive regions were successfully performed on 23 of the 25 available samples. Both LINE1 and ALU-Yb8 mean methylation extents in peripheral blood demonstrated smaller ranges from 70% to 85% and 83% to 88%, respectively, but had wider ranges in saliva samples of 53% to 84% and 63% to 86%, respectively. We, therefore, compared differences in the medians of LINE1 and ALU-Yb8 methylation extent between donors and recipients stratified by sample type. Only LINE1 methylation extent in peripheral blood was significantly greater (Figure 1) among recipients than donor individuals, although we note that the small number of samples within the groups limits power to detect differences.

**Figure 2:**
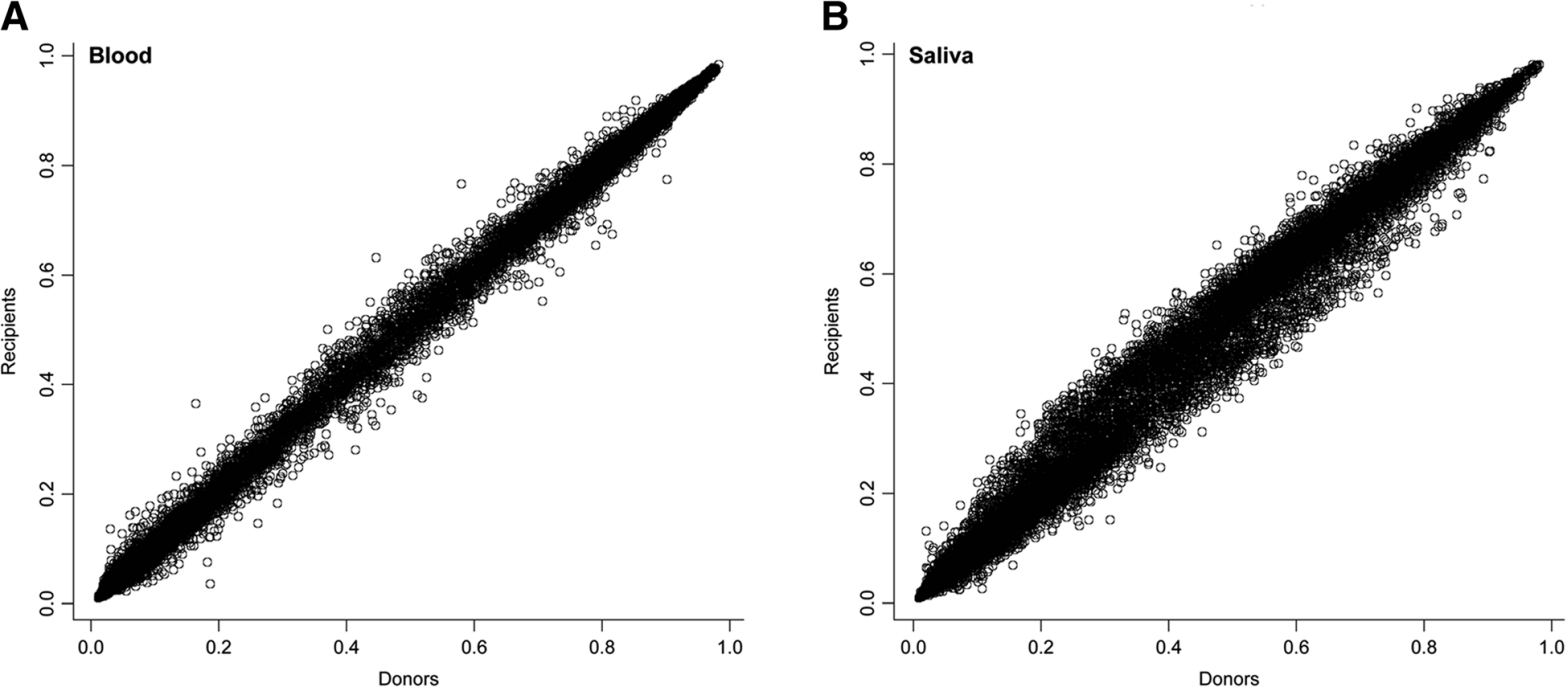
**Scatterplots of autosomal CpG loci DNA methylation relationships between all donors and recipient pairs.** Donors methylation values are depicted on the X-axes, recipient on the Y-axes in **(A)** peripheral blood and **(B)** saliva.

To more broadly characterize differences across the epigenome in donor compared to recipient children, we utilized the Illumina Infinium Methylation27 Beadarray to assess genome-wide patterns of DNA methylation. Figure 2 depicts the mean methylation of all donors (X-axes) compared to all recipients (Y-axes) in blood and saliva at all 26,486 autosomal loci examined using the arrays. All genome-wide analyses performed were stratified by sample type as it is well established that the pattern of DNA methylation is highly cell and tissue specific [35]. In peripheral blood there was strikingly little variation between methylation genome-wide between donors and recipients. A greater extent of variation was observed in the saliva samples, particularly in those samples with beta values between 0.2 and 0.6, but less so among loci exhibiting nearly complete hypo- (beta = 0) or hyper- (beta = 1) methylation.

**Figure 3:**
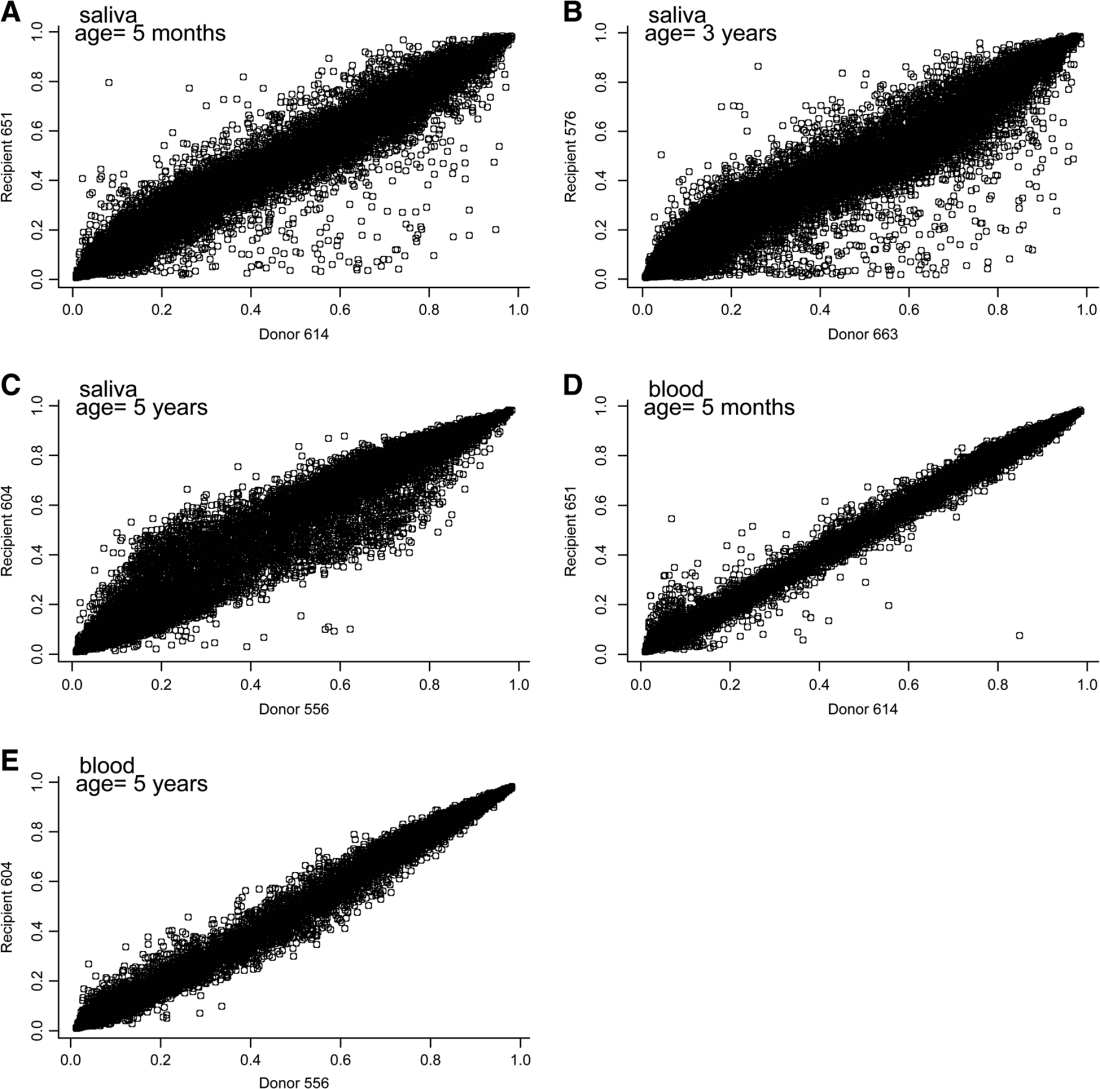
**Scatterplots of autosomal CpG loci DNA methylation relationships between individual donor and recipient twin pairs.** Panels **A-C** each depict a twin pair in saliva with donor twins on the X-axes and recipient twins on the Y-axes, while Panels **D-E** depict twin pair comparisons of methylation in peripheral blood samples. The ages of the children at the time of sample collection are provided on each panel.

Variation in the methylation at these loci could potentially be arising from other sources, and so we next examined the relationship between genome-wide methylation among the available twin pairs in the study, stratified by sample type. In this case, only ten samples, or five pairs, satisfied our stringent quality control criteria. Figure 3 depicts these individual comparisons, and again, saliva samples showed greater variation between donor and recipient individuals. Particularly, in saliva samples there appeared to be deviations from the diagonal with recipients showing greater methylation of loci whose methylation beta in donors is between 0.2 to 0.3 and lesser methylation of loci whose methylation beta in donors is between 0.7 to 0.9. In general, the blood samples showed less variability between donors and recipients, although in the comparison of the five month old twins’ blood samples, there appeared to be a number of variable loci at the lowest levels of methylation. In addition to the age of the child when the samples were obtained, the gestational age at which the fetal laser surgery was performed could also affect the variability in methylation between donors and recipients, considering that infants with earlier surgery may have a longer period to equalize their methylation patterns. We attempted to address this as well as the issue of age at sampling by examining the number of loci whose methylation beta differed between donor and recipient pair by greater than 0.2 or less than −0.2, which corresponds to greater than three standard deviations of the mean difference in saliva. The number of loci with these extreme changes is tabulated in Table 2. We then compared the subjects’ age when sampled, as well as their gestational age at fetal surgery. There was a trend for a greater number of highly differential loci among subjects with a later age at surgery and with increasing age at the time of sampling, with both correlations >0.85, although the sample sizes here limited formal examinations of inference, particularly for blood samples.

**Table 2:** **Number of loci with large differences in methylation between donors and recipients**, **and their relationship to surgery age and current age**

**Sample type**	**Donor ID**	**Recipient ID**	**Total number of loci with methylation difference** >**0**.**2 or** <−**0**.**2**	**Gestational age at laser surgery** (**weeks**)	**Current age when sampled** (**years**)
Saliva	614	651	296	15.86	0.42
Saliva	663	576	551	20.86	3
Saliva	556	604	540	18.14	5
Blood	614	651	24	15.86	0.42
Blood	556	604	6	18.14	5

Pearson correlation between age of fetal surgery and number of loci with a large difference in saliva is 0.86 (*P* <0.3). Pearson correlation between the age when the sample was obtained and the number of loci with large difference in saliva is 0.88 (*P* <0.3).

**Figure 4:**
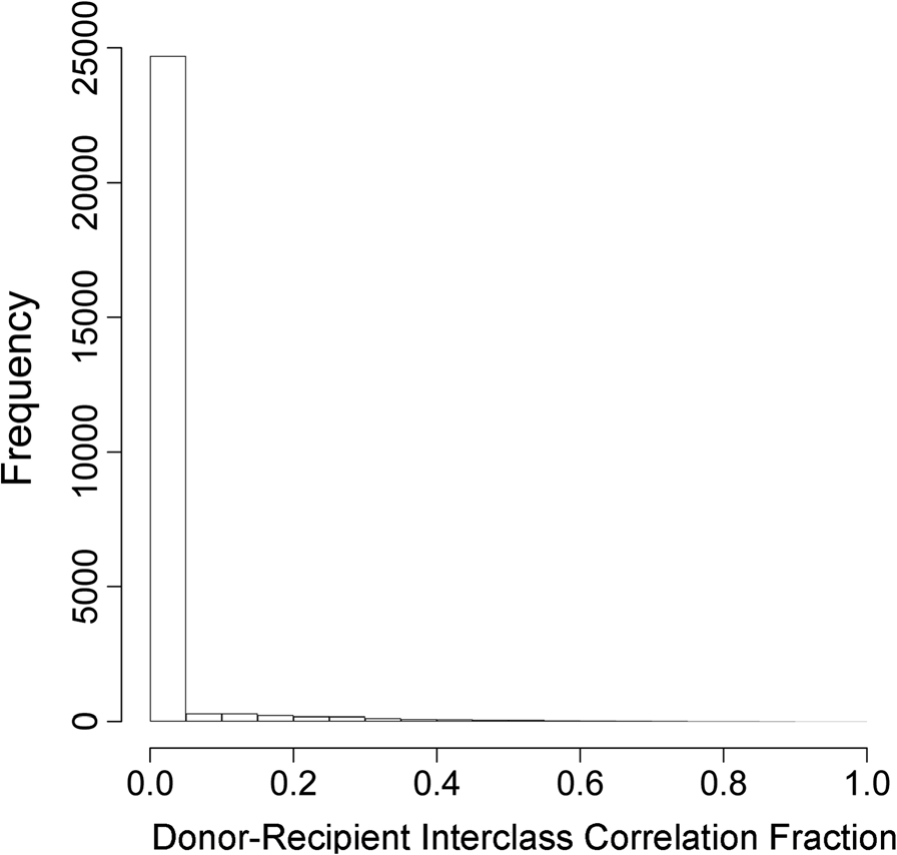
**Histograms of the distribution of the interclass correlations.** Values depict the fraction of variation explained by donor-recipient status resulting from linear mixed effects models examining the sources of variability of methylation across all 26,486 autosomal loci.

To formally examine the types of loci demonstrating variability between donors and recipients, we employed a linear mixed effects model, including random effects for pair membership and donor-recipient status within a pair. This model allowed us to include all of our data in a single model, thereby improving power. We used these models to calculate the interclass correlation coefficient (ICC) for each loci. This provides an estimate of the variability in methylation related to donor-recipient status compared to the overall variability of methylation. Loci with an ICC closer to 1 would be those loci whose variability could be most explained by donor-recipient status. Figure 4 depicts the distribution of donor-recipient ICC values. It would appear that the majority of variability in methylation is not explained by donor-recipient status, although there were a small number of loci with ICC >0.8. Examples of some of these loci are provided in Additional file 1: Figure S1. Using a gene-set enrichment analysis approach, we found that loci with high donor-recipient ICC were significantly more likely (*P* = 0.0002) to be polycomb group target genes (PcG) [36,37] but were not significantly enriched for genes within CpG islands (*P* = 0.14). To assess if the variability of methylation observed in both blood and saliva samples may be attributable to differential distributions of blood cell types within the samples examined, we ranked all of the loci by their association with leukocyte subsets defined previously [38,39] and examined the values of the donor-recipient ICC by this ranking [see Additional file 1: Figure S2]. Among loci most considered differentially methylated across blood cell types, we found low to moderate ICC values, while those loci exhibiting ICC values >0.8 were not highly ranked as differentially methylated across blood cell types.

## Discussion

Using a unique population of TTTS survivors, we have identified subtle but potentially important variability in DNA methylation influenced by the physiologic environment during development. Epigenetic mechanisms, such as DNA methylation, are clearly important in mediating the developmental origins of lifelong health and disease. By taking advantage of this unique and valuable cohort, we have been able to demonstrate that regardless of tissue type, developmentally critical genes controlled by polycomb group transcription factors and modifiers are being targeted for altered DNA methylation. These findings provide additional evidence that through epigenetic alterations the intrauterine environment can affect cellular function beyond the developmental period. Additionally, our results may provide important insights into the mechanisms leading to the potential morbidities in survivors of TTTS, which are growing in number due to advances in successful surgical interventions of this previously mortal condition [25].

We identified subtle differences in global markers of DNA methylation, specifically the finding of increased methylation of the LINE1 element among recipients. These results are in line with recent findings that nutritional factors can influence global markers of DNA methylation. For example, fortification of the diet with folate has been correlated with increased peripheral blood LINE1 methylation in adults [40] although in a folate-replete population, little difference in LINE1 methylation was observed in infant cord blood based upon maternal intake of methyl donors [41]. LINE1 hypomethylation in cord blood has been associated with extremes in birth weight (both high and low) and with prematurity [42]. In adults, alterations in LINE1 methylation have been associated with various health outcomes including cancers and cardiovascular disease [43] but the mechanism through which non-target tissue alterations of methylation of these global markers leads to disease is not understood [43]. To date, there have been little data regarding any relationships with this marker in childhood or adolescent populations, making this study unique.

Overall, our examination revealed low levels of variability of DNA methylation between donors and recipients at the locus-specific, genome-wide level. However, when individual twin pairs were examined, these differences were clearer. The saliva samples showed greater variability between donors and recipients. Variability did not appear to occur consistently across all CpG sites and was lowest at the loci with beta values at the extremes (0 and 1) and at the midpoint (0.5). This would represent those loci with complete or nearly complete hypo- and hypermethylation. Loci with intermediate methylation potentially represent imprinted regions. Interestingly, in blood, comparing donor and recipient methylation in a five-month-old infant, there are a number of variable loci at the lowest levels of methylation. Importantly, since blood as well as saliva [44] represents a mixture of cell types, this may represent changes in the proportion of specific blood cell types characterized by methylation (or lack of methylation) at specific loci, such as the FOXP3 locus, which characterizes T-regulatory cells [45]. In fact, recent studies have shown that much of the variability identified in peripheral blood DNA methylation profiles represents variation in the underlying proportion of blood cell types [38,39]. We examined if the loci with the greatest interclass correlation by donor-recipient status would be considered differentially methylated across blood cell subtypes, and saw little evidence. Yet, the data on blood cell differentially methylated regions were based on adult signatures and may not be completely comparable to the data examined here on infants and young children, thus making more specific examinations worthy of further explorations.

Our observation that there are a greater number of severely altered loci among twin pairs with later fetoscopic surgery is intriguing, although preliminary. From these findings, we could speculate that differences in methylation are more common, the longer the uneven placental environment exists, as infants undergoing surgery at the latest time points (18 to 20 weeks) had nearly twice as many loci with highly divergent methylation in their saliva samples as those with surgery at 15 weeks. Even with surgery at 15 weeks, there are a substantial number of divergent loci, suggesting that some of these effects may be occurring early in development, even before laser treatment or TTTS diagnosis, is possible. Coincidentally, the infants with early surgery were also the youngest pair, while those with later surgery were the oldest. It is, therefore, possible that these effects are derived postnatally. Yet, considering that these twins are being raised in similar environments postnatally, there remains strong reason to believe that early correction of the discordant uteroplacental environment may limit epigenetic divergence.

We observed the greatest divergence amongst loci considered polycomb group targets. Polycomb group (PcG) genes are a family of developmentally important genes, which play a role in chromatin post-translational modification and remodeling and which are responsible for silencing key developmentally regulated genes [46,47]. The polycomb group family members are responsible for targeting developmentally regulated genes such as the HOX family and those involved in cellular differentiation, and evidence is accumulating that DNA hypermethylation is observed in many cancers at the sites of polycomb-mediated gene repression in embryonic cells [48]. Our finding that the genes exhibiting the greatest variation related to the donor recipient status of the child are over-represented by PcG targets highlights the potential importance of this variability. The role that such variability may play in downstream disease risk and morbidity deserves further study.

The strengths of this study include the unique study population and the opportunity to examine twin pairs in the context of TTTS. We employed state-of-the-art genome-wide methodologies for the assessment of DNA methylation. Further, we utilized appropriate and powerful statistical methodologies to quality control and analyze the resulting data. We recognize the limitation of sample size in our analyses, and have carefully constructed our analyses and our interpretations with this limitation in mind. Particularly, this is why we have not sought to identify genome-wide significant loci or genes with methylation associated with donor-recipient status or other features of the children involved. Instead, we devised strategies to broadly describe key features of those genes targeted for disruption by this condition. We also recognize that there is some potential confounding by genome in those samples from individuals whose twin did not survive to be included in this analysis. In addition, due to the small sample size, we could not fully address the modification of these effects by age or gender. Future studies are needed to identify more precisely those genes targeted for alteration, to understand better how various factors impact these alterations, and to link those alterations with downstream outcomes in the survivors of TTTS.

## Conclusions

Overall, this study provides an intriguing, albeit preliminary, description of the epigenomic landscape of childhood survivors of TTTS. Studies of this condition and its consequences present a unique opportunity to consider more carefully mechanisms involved in the developmental origins of health and disease. Future evaluation of these cohorts holds great promise to identify the molecular basis of downstream health consequences faced by children following successful intervention for TTTS.

## Methods

### Study population

Informed consent was obtained for all individuals involved in this study under the review of the Institutional Review Board of Rhode Island Hospital. Subjects were identified by review of medical records of all patients undergoing endoscopic laser ablation of placental vessels for severe TTTS at the Fetal Treatment Program of Rhode Island Hospital since 2000. Families with at least one twin who survived beyond the neonatal period were contacted, and were asked to obtain, for each surviving twin, saliva samples as well as peripheral venous blood at the child’s next well patient visit with their pediatrician. Saliva was obtained with the Oragene Discover system for assisted collection (DNA Genotek, Kanata, Ontario, Canada), and peripheral blood (approximately 2 ml) was collected in an ethylenediaminetetraacetic acid (EDTA) tube. A total of 14 children, including 8 pairs, participated, with 11 blood samples and 14 saliva samples available for analysis. All dual survivors in this study as well as four of the six single survivors were stage III or IV, while the remaining two single survivors were stage II. Growth restriction was observed in two of the donor infants, who experienced catch-up growth following successful laser surgery.

### DNA extraction and modification

DNA was extracted from the whole blood samples using the QIAmp DNA Mini Kit (Qiagen, Inc., Valencia, CA, USA) following the manufacturer’s protocols, and was extracted from the saliva samples using reagents and protocols provided with the Oragene Discover system. Purified DNA was quantified using a ND-1000 spectrophotometer (Nanodrop, Wilmington, DE, USA). All DNA samples (1 μg) were bisulfite-modified using the EZ DNA Methylation Kit (Zymo Research, Irvine, CA, USA ) and stored at −20°C.

### Bisulfite pyrosequencing for Alu-Yb8 and LINE1 methylation

The extent of methylation of the Alu-Yb8 and LINE1 repetitive elements was used as a marker of global methylation and was assessed in all samples available using bisulfite pyrosequencing as previously described [49] on the Pyromark MD Pyrosequencer. Methylation extent was calculated as the mean methylation across four positions in the LINE1 region and five positions in the Alu-Yb8 region. Pyrosequencing reactions were performed in triplicate for each sample and the mean of the triplicates was used in all analyses. All pyrosequencing reactions also included bisulfite modification assessments. If any sample demonstrated less that 97% modification efficiency, that sample was re-modified and all reactions were repeated.

### Array-based DNA methylation assessments

To examine gene-related CpG methylation, methylation was measured at 27,578 CpG loci using the Infinium HumanMethylation27 Bead Array (Illumina, San Diego, CA, USA). The microarrays were processed at the University of Californa at San Francisco Institute for Human Genetics Genomic Core Facility, following standard protocols. The methylation status for each individual CpG locus was calculated as the ratio of fluorescent signals (β = Max(M,0)/[Max(M,0)+Max(U,0) + 100]), ranging from 0 to 1, using the average probe intensity for the methylated (M) and unmethylated (U) alleles. Beta (β) = 1 indicates complete methylation; β = 0 represents no methylation. The data were assembled using BeadStudio methylation software (Illumina), without normalization according to the manufacturer’s instructions. We used array control probes to assess the quality of our samples and to evaluate potential problems, such as poor bisulfite conversion or color-specific issues for each array, and found these quality control probes to have similar distributions across all samples. Any samples with >25% of CpG loci having a detection *P*-value >0.05, or any loci demonstrating a detection *P*-value >0.05 in more than 20% of samples was removed [50]. All CpG loci on X and Y chromosomes were excluded from the analysis, to avoid gender-specific methylation bias, leaving a final 26,486 autosomal CpG loci representing 13,890 unique genes in a total of 23 samples. We also performed a principle components analysis of the array data, and examined the association between the top four principle components and beadchip, to assess if the predominant variation across samples was based on technical characteristics and found no associations.

We, and others, have previously demonstrated that methylation of CpG loci detected through BeadArray platforms can be reliably replicated using alternative detection techniques including pyrosequencing, mass array analysis and quantitative methylation-specific PCR [50-56].

### Statistical analysis

The extents of methylation of LINE1 and ALU-Yb8 were compared between donors and recipients, stratified by sample type, using the nonparametric Mann–Whitney U test in order to limit the influence of outlier points.

For the array based DNA methylation data, we used the following random effects model to account for the data structure:

$$Y_{\mathrm{ijkl}}=\alpha +u_{j}+v_{\mathrm{jk}}+\epsilon_{\mathrm{ijkl}}$$

where *Y*_*ijkl*_ represents the methylation *M*-value (that is, log2 ratio of the intensities of methylated probe versus unmethylated probe) [57], for individual *i* = 1, 2, …, 14, pair *j* = 1, 2, …, 8, *k* an index for donor/recipient status, *l* an index for sample type (that is, blood or saliva); α is the overall intercept, *u*_*j*_ and *v*_*jk*_ are random effects associated with pair and donor/recipient status within pair, and *ϵ*_*ijkl*_ is the error term. Using standard linear mixed effects model formulation, we assume the random effects and residual errors are independent and normally distributed: $u_{j}∼N\left(0,\sigma_{1}^{2}\right);v_{\mathrm{jk}}∼N\left(0,\sigma_{2}^{2}\right);$ and $\epsilon_{\mathrm{ijkl}}∼N\left(0,\sigma_{3}^{2}\right)$, yielding three variance components in the model (that is, *σ*_1_^2^, *σ*_2_^2^ and *σ*_3_^2^). The random effect variance *σ*_2_^2^ reflects the variation in methylation between donors and recipients. We used the intra-class correlation coefficient to understand the stability of DNA methylation between donors and recipients. We defined the ICC here to be:

$$\mathrm{ICC}=\frac{\sigma_{2}^{2}}{\sigma_{1}^{2}+\sigma_{2}^{2}+\sigma_{3}^{2}},0\leq \mathrm{ICC}\leq 1$$

with values approaching 1 signifying that the predominant source of variability in methylation was between donors and recipients. Model (1) was fit independently to all autosomal CpG loci that passed quality assurance and quality control procedures. To avoid potential biases in assessing the stability of methylation markers between donors and recipients, models were adjusted for patient age at the time of sample collection.

To evaluate the biological relevance of the obtained ICCs, we examined the association between ICC values and characteristics of the methylation markers. In particular, Wilcoxon rank-sum tests were used to investigate the association between ICC values and polycomb group target gene (PcG) [36,37] associated loci as well as CpG Island associated loci.

To examine if the differences in methylation between donor and recipients represent differences in the underlying proportion of blood cell types within the sample, we ranked the loci on the arrays based on their prior association with blood leukocyte methylation [38,39] which has defined blood cell specific differentially methylated regions. We then examined the current ICC value by leukocyte DMR rank.

All analyses were carried out using the R statistical package, version 2.13 (http://www.r-project.org/).

## Competing interests

The authors declare that they have no competing interests.

## Authors’ contributions

CJM, CMB, JFP and FL designed the study. DWS and FL were responsible for subject recruitment and sample ascertainment. CJM was responsible for the molecular analyses. DCK and CJM were responsible for the statistical analyses. CJM, DCK, DWS, CMB, JFP and FL wrote and edited the manuscript. All authors read and approved the final version of the manuscript.

## Supplementary Material

Additional file 1: Figure S1 Examples of three loci demonstrating high donor-recipient interclass correlation values, each suggesting that >80% of their variability is explained by donor-recipient status. Red lines denote the mean methylation within each group. **Figure S2**. Value of the donor-recipient interclass correlation (ICC2) values for all loci ranked by their DMR status, based on prior studies of leukocyte subset DNA methylation. Click here for file
