# Supplementary material for: Developmental genes targeted for epigenetic variation between twin-twin transfusion syndrome children
Source: Clin Epigenetics. 2013 Oct 3;5(1):18. doi: 10.1186/1868-7083-5-18 (PMC4016001; doi:10.1186/1868-7083-5-18)
Supplement: Additional file 1: Figure S1 — Examples of three loci demonstrating high donor-recipient interclass correlation values, each suggesting that >80% of their variability is explained by donor-recipient status. Red lines denote the mean methylation within each group. Figure S2. Value of the donor-recipient interclass correlation (ICC2) values for all loci ranked by their DMR status, based on prior studies of leukocyte subset DNA methylation. [file 1868-7083-5-18-S1.pdf]

Supplementary Figure 1.

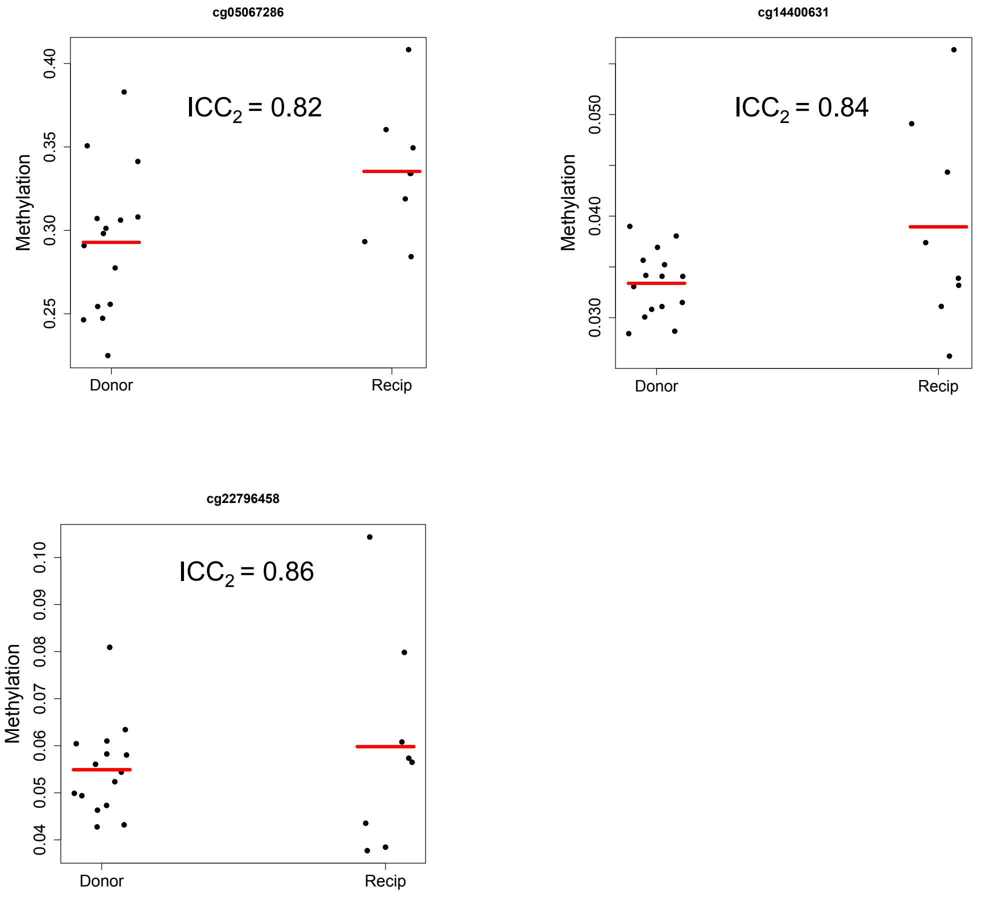

Supplementary Figure 1. Examples of 3 loci demonstrating high donor-recipient interclass correlation values, each suggesting that >80% of their variability is explained by donor-recipient status. Red lines denote the mean methylation within each group.

Supplementary Figure 2

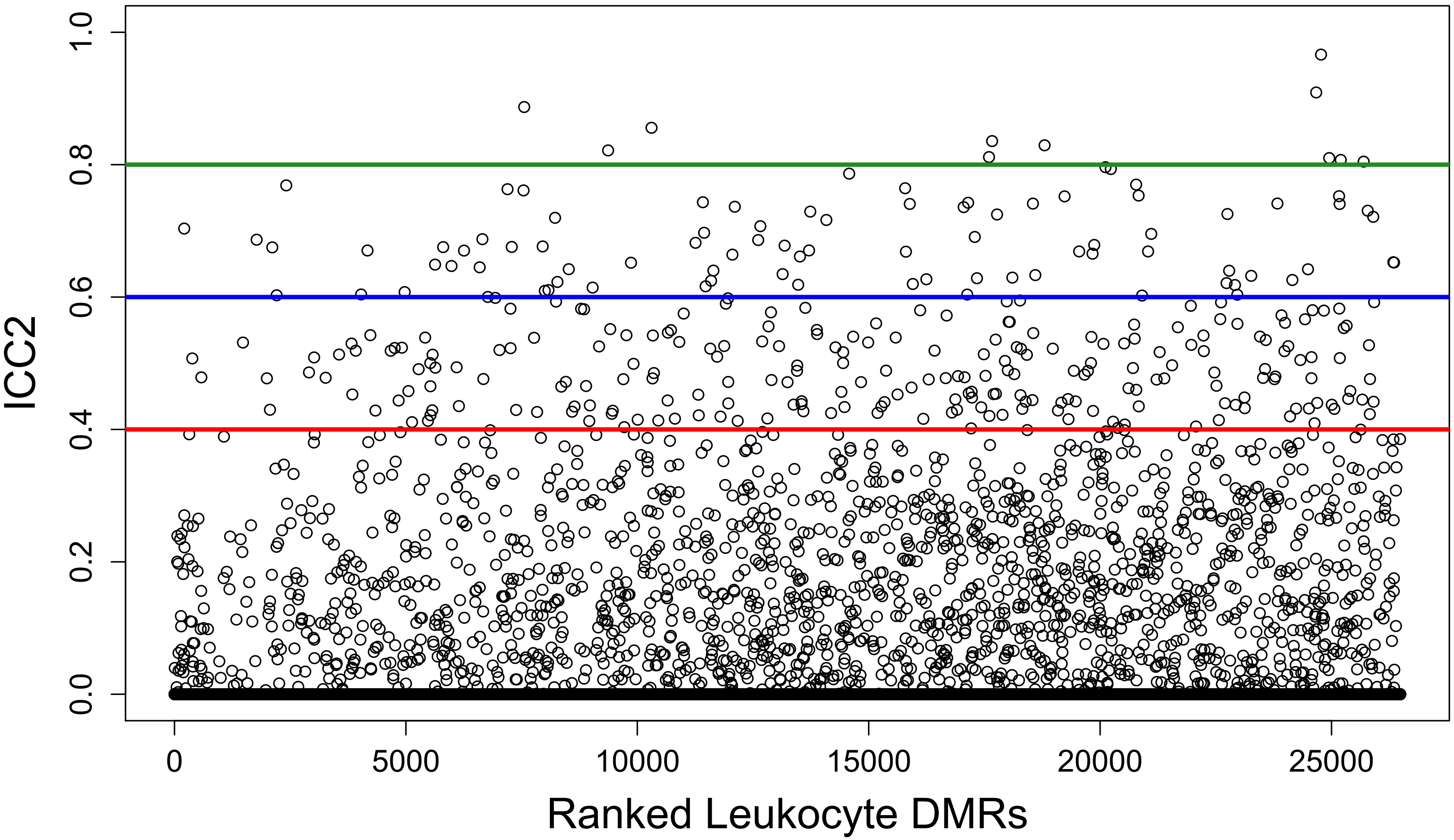

Supplementary Figure 2. Value of the donor-recipient interclass correlation (ICC_2) values for all loci ranked by their DMR status, based on prior studies of leukocyte subset DNA methylation.
